# Disturbed Ratios between Essential and Toxic Trace Elements as Potential Biomarkers of Acute Ischemic Stroke

**DOI:** 10.3390/nu15061434

**Published:** 2023-03-16

**Authors:** Anna Mirończuk, Katarzyna Kapica-Topczewska, Katarzyna Socha, Jolanta Soroczyńska, Jacek Jamiołkowski, Monika Chorąży, Agata Czarnowska, Agnieszka Mitrosz, Alina Kułakowska, Jan Kochanowicz

**Affiliations:** 1 Department of Neurology, Medical University of Bialystok, M. Skłodowskiej-Curie 24a Street, 15-276 Białystok, Poland; 2 Department of Bromatology, Faculty of Pharmacy with the Division of Laboratory Medicine, Medical University of Białystok, Mickiewicza 2D Street, 15-222 Białystok, Poland; 3 Department of Population Medicine and Lifestyle Diseases Prevention, Medical University of Bialystok, M. Skłodowskiej-Curie 24a Street, 15-276 Białystok, Poland

**Keywords:** essential trace elements, heavy metals, cadmium, lead, ischemic stroke, atherosclerosis

## Abstract

Background: Cadmium (Cd) and lead (Pb) are known to be two of the metal contaminants that pose the greatest potential threat to human health. The purpose of this research study was to compare the levels of toxic metals (Cd, Pb) in patients with acute ischemic stroke (AIS), with a control group in Podlaskie Voivodeship, Poland. The study also aimed to assess the correlations between toxic metals and clinical data in AIS patients, and to assess the potential effect of smoking. Materials and methods: The levels of mineral components in the collected blood samples were assessed by means of atomic absorption spectrometry (AAS). Results: The Cd blood concentration was significantly higher in AIS patients as compared to the control group. We found that the molar ratios of Cd/Zn and Cd/Pb were significantly higher (*p* < 0.001; *p* < 0.001, respectively), when the molar ratios of Se/Pb, Se/Cd, and Cu/Cd were significantly lower (*p* = 0.01; *p* < 0.001; *p* < 0.001, respectively), in AIS patients as compared to control subjects. However, there were no considerable fluctuations in relation to the blood Pb concentration or molar ratios of Zn/Pb and Cu/Pb between our AIS patients and the control group. We also found that patients with internal carotid artery (ICA) atherosclerosis, particularly those with 20–50% ICA stenosis, had higher concentrations of Cd and Cd/Zn, but lower Cu/Cd and Se/Cd molar ratios. In the course of our analysis, we observed that current smokers among AIS patients had significantly higher blood-Cd concentrations, Cd/Zn and Cd/Pb molar ratios, and hemoglobin levels, but significantly lower HDL-C concentrations, Se/Cd, and Cu/Cd molar ratios. Conclusions: Our research has shown that the disruption of the metal balance plays a crucial role in the pathogenesis of AIS. Furthermore, our results broaden those of previous studies on the exposure to Cd and Pb as risk factors for AIS. Further investigations are necessary to examine the probable mechanisms of Cd and Pb in the onset of ischemic stroke. The Cd/Zn molar ratio may be a useful biomarker of atherosclerosis in AIS patients. An accurate assessment of changes in the molar ratios of essential and toxic trace elements could serve as a valuable indicator of the nutritional status and levels of oxidative stress in AIS patients. It is critical to investigate the potential role of exposure to metal mixtures in AIS, due to its public health implications.

## 1. Introduction

There is growing concern regarding the potential role of unorthodox threatening factors, such as exposure to air pollution, environmental chemicals, heavy metals, and persistent organic pollutants regarding cardiovascular disease development (CVD) [1,2]. These factors, alongside traditional risk factors such as obesity, diabetes, hypertension and dyslipidemia, have been considered as potential CVD contributors.

According to the study by Feigin et al. [3], behavioral factors such as smoking, suboptimal nutritional choices, and inadequate physical activity account for a significant proportion (47.0%) of stroke burden. However, environmental risks, including air pollution and exposure to toxic metals, contribute an additional 37.8%. Multiple studies have suggested that exposure to various metals and metalloids can adversely affect immune function and increase the risk of CVD [4]. Environmental pollution is generally considered the main factor contributing to human exposure to heavy metals. Cadmium (Cd), arsenic (As), lead (Pb), and mercury (Hg), among others, are usually highlighted because of their high toxicity. Furthermore, Cd and Pb have been identified as two of the metal contaminants that pose the greatest potential harm and risk to human health [5]. The study involved patients living in the Green Lungs of Poland, situated in the northeastern region of the country. Podlaskie Voivodeship, Poland, is an ecological region, predominantly agricultural, with minimal industrialization and no significant heavy industry. Thus, the primary sources of heavy metal exposure are contaminated food due to pollution, and tobacco smoking [6,7]. Cd and Pb are significant environmental pollutants in Podlaskie Voivodeship, due to their prevalence, despite having relatively low concentrations [8,9]. These toxic trace elements pose a significant threat to the environment as they can easily contaminate food sources, leading to the exceedance of maximum acceptable food content levels. On the other hand, exceedances of arsenic and mercury pollution are rare, even in local fish populations in the region [10]. Consistent with the systematic review by Bao et al. [5], the effect of arsenic and mercury on stroke risk was found to be reduced.

Unbalanced levels of metals in the body have been shown to disrupt homeostasis and contribute to the progression of various diseases, including ischemic stroke [5,11,12,13,14,15]. However, the indication regarding the connection between exposure to heavy metals and stroke risk remains inconclusive.

Exposure to trace elements such as Pb and Cd for the general public occurs through inhalation, dermal absorption, and long-term consumption of food products and contaminated water, as well as exposure to soil, dust and fumes, industrial materials, consumer products and ambient air [16]. Heavy metal toxicity has been linked to a range of conditions, including neurological and neurodegenerative disorders, cancer, bone and kidney diseases, and autoimmune disorders [7,17,18]. According to Renu et al.’s study, heavy-metal-induced liver toxicity is often accompanied by inflammation that is triggered by the activation of proinflammatory cytokines, TNF-alpha, and the ERK and MAPK pathways [19]. These toxic metals can disrupt the balance of essential trace elements in the body and interfere with their physiological and biochemical processes [19,20].

Cadmium (Cd) tends to accumulate primarily in the kidneys (50%), liver (15%), and muscles (20%) in humans. It is also found in high concentrations in erythrocytes, while its concentration in plasma is very low [21]. The possible mechanism of Cd neurotoxicity is the induction of oxidative stress, the disruption of the activity of enzymes essential for the proper functioning of the nervous system, and the destruction of the homeostasis of bioelements in the brain [22]. Comprehensive evaluations and combined analyses on the relationship between Cd and CVD have included studies on stroke, taking into account smoking habits [23,24]. Current smokers have approximately twice as much Cd in their kidneys and urine, and three to four times as much Cd in their blood, compared to never-smokers [25,26]. Numerous medical investigations have determined that it is inclined to accumulate in the vasculature’s intima. Cd has been associated with the formation of carotid plaques [27], carotid atherosclerosis [23,28,29,30,31], increased risks of CVD [14,32,32,33,34,35,36] and stroke [5,11,14,25,32,37,38,39,40,41,42,43], hypertension [44,45,46], peripheral arterial disease [47], myocardial infarction [48,49] and inflammation [50,51]. It has also been proved that exposure to Cd and obesity may have significant synergistic results on the onset of diabetes [52,53]. All the factors mentioned above are involved in stroke risk. However, data directly linking Cd exposure to the risk of ischemic stroke are limited, and the findings are contradictory.

Lead is mainly taken in by respiration through the airways or digestion through the digestive system, with subsequent accumulation in bone, blood, and organs such as the brain, kidneys, and liver [54]. Pb has a half-life of several weeks in the circulatory system and nearly two years in the brain. It can accumulate in bones, serving as a continuous internal source of Pb that can leach out over time and affect the vascular endothelium and other tissues [18,55]. The central nervous system is highly susceptible to Pb toxicity. Research has proven that Pb-triggered endothelial dysfunction may increase the risk of CVD, cerebral atherosclerosis and neurodegenerative diseases [56]. Most studies analyzing the effects of Pb on the general population have focused on cardiovascular morbidity. The increased levels of Pb are associated with an increased risk of heart disease, atherosclerosis, hypertension, and cardiac disease, due to cellular signaling and atherosclerotic changes, inflammation, and dysregulation of lipid metabolism [57,58].

The detection of heavy metals has recently become a key focus in medical research. There is growing evidence that suggests a positive correlation between certain environmental pollutants and the incidence of stroke. A meta-analysis of 37 independent investigations reported a linear dose–response relationship between exposure to Pb and Cd and CVD risk, particularly stroke [32]. Furthermore, a systematic review conducted by Bao et al. highlights the fact that long-term exposure to lead and copper is associated with an increased risk of stroke [5]. The purpose of this research study was to compare the levels of toxic trace elements (Pb, Cd) in acute-ischemic-stroke (AIS) patients to those of a control group in Podlaskie Voivodeship, Poland. The study also aimed to assess the correlations between toxic metals and clinical data in stroke patients and evaluate the potential effect of smoking. Based on our previous research [59], which investigated the concentrations of essential trace elements (selenium, zinc, copper), this study examined the relationship between toxic metals and essential trace elements in AIS patients. At present time, few, if any, detailed investigations have been conducted to analyze the status of toxic metals in AIS patients in a Polish population.

## 2. Materials and Methods

This medical research was carried out from January 2019 to November 2021 at the Medical University of Bialystok (MUB) in the Department of Neurology. A total of 187 AIS patients were enrolled in the study, including 85 patients who received intravenous thrombolysis and/or mechanical thrombectomy and 102 patients who received conservative treatment. The criteria for inclusion in the study were specified as follows: age between 18 and 85 years at the time of enrolment, hospitalization within 24 h of the onset of neurological symptoms, computed tomography (CT), and/or magnetic resonance imaging (MRI) to estimate the magnitude of the infarction and eliminate intracranial and subarachnoid hemorrhage and tumors. Individuals who participated in the study were selected 2–5 days after the onset of clinical signs. Exclusion criteria comprised: previous stroke or ischemic stroke of undetermined etiology (UD) according to the TOAST classification (Trial of Org 10,172 in Acute Stroke Treatment) [60], acute surgical and traumatic diseases, myocardial infarction, or acute and contagious infections and inflammations within the past month. Furthermore, advanced heart failure, autoimmune diseases (rheumatic disease), stage 5 chronic kidney disease, liver failure, cancer, recent intake of mineral supplements in the last 3 months, metal implants, or hormonal therapy.

A group of 94 control subjects with no history of stroke or chronic cerebrovascular disease was selected from individuals who voluntarily contacted the Department of Bromatology at the Medical University of Bialystok. Demographic, clinical, and cardiovascular risk factors, including arterial hypertension, smoking status (never, former, or current smoker), diabetes, excessive alcohol consumption, abnormal levels of lipoproteins, previous cardiac disorders, atrial fibrillation, and a history of previous stroke, as well as a history of medication and laboratory records, were evaluated. Neurological status was estimated using the National Institutes of Health Stroke Scale (NIHSS) during admission and discharge [61] and the modified Rankin Scale (mRS) [62] on discharge. The etiology of ischemic stroke was established using the TOAST classification (Trial of Org 10,172 in Acute Stroke Treatment) [60] and was classified into three categories: cardioembolism (CE), large artery atherosclerosis (LAA), and small vessel occlusion (SVO). Findings from neurological assessment and various imaging techniques such as CT or MRI of the brain, B-mode external ultrasound carotid imaging, echocardiography, head and/or neck CT angiography, and 12-channel ECG were used as the basis for the evaluation. Smoking status was classified as never-smokers or smokers, including former (stopped smoking ≤15 years ago) or current smokers. Body mass index (BMI) was determined by the patient’s weight expressed in kilograms divided by height in meters squared, with values <25.0 or ≥25.0 considered normal weight or overweight/obese, respectively.

The protocol used for this research was verified and accepted by the Ethics Committee at the Medical University of Bialystok (reference number R-I-002/276/2018). All study participants or their authorized representatives expressed a written and informed consent prior to the collection of blood specimens and the updating of clinical data from medical records.

### 2.1. Blood Sample Collection and Analysis

Approximately 8 mL of blood samples were taken from each study participant using a vacuum blood-collection system with a clot activator (Becton Dickinson, France). The samples were collected within 3 to 5 days of the neurological symptoms’ onset and were processed by centrifuging at 2500× *g* rpm for 10 min (MPW M-Diagnostic, Med. Instruments, Warsaw, Poland). Samples of whole blood and serum were subsequently preserved at −20 °C in the Department of Bromatology (MUB). All reagents and chemicals utilized in the study were of high quality and suitable for spectral analysis.

A total of 200 µL of whole blood were taken into Eppendorf tubes, and 800 µL of 1 mol/L of nitric acid (Merck, Darmstadt, Germany) and 200 µL of 1% Triton X-100 (Sigma, Taufkirchen, Germany) were added. The samples were vortexed and centrifuged for 10 min at 2500× *g* rpm. The supernatant was then further diluted 3 times with 0.1 mol/L HNO_3_. The concentrations of Cd and Pb in the blood samples were determined by applying atomic absorption spectrometry (AAS) with flameless atomization in a graphite cuvette and Zeeman background rectification using the following wavelengths: 228.8 nm and 283.3 nm, respectively (Z-2000, Hitachi High-Technologies Corporation, Tokyo, Japan). During the determination of Cd, the following analytical program (start/end temperature) was used: drying 80/140 °C, ashing 140/450 °C, atomization 1600/1600 °C and graphite cuvette cleaning 1800/1800 °C. During Pb determination, the following analytical program was used: drying 80/140 °C, ashing 140/600 °C, atomization 2400/2400 °C and graphite cuvette cleaning 2700/2700 °C. Matrix modifiers, 1000 mg/L palladium nitrite (Merck, Darmstadt, Germany) for Cd and 0.5% ammonium dihydrogen phosphate (Sigma, Taufkirchen, Germany) for Pb, were then added to eliminate interferences from the matrix. Calibration solutions were prepared using standard solutions of Cd and Pb with a concentration of 1 g/L (Merck, Darmstadt, Germany). The detection limits for Cd and Pb were 0.053 μg/L and 0.45 μg/L, respectively. The precision of Cd and Pb determinations was verified using certified reference material, Seronorm Trace Elements Whole Blood L-2 (Sero AS, Hvalstad, Norway). On average, the percent recovery values reported for the analytical methods used to determine the content of Cd and Pb in the reference material were 98.4% and 103.2%, respectively, and the precision of the methods was 3.4% and 2.5%, respectively. The concentrations of Cu (copper) and Se (selenium) in serum were also determined by atomic absorption spectrometry with electrothermal atomization using a Zeeman background correction, as previously described [59]. The concentration of Zn (zinc) in serum was determined using the same method with air-acetylene flame atomization [59]. The reliability of these methods was verified using certified reference material from human serum (Seronorm trace elements, Serum L-1, SeroA, Billingstad, Norway). The conclusions of the quality control evaluations were consistent with the reference values. Biochemical assays were performed using protocol standards, and the Cd, Pb, and Se values are expressed in μg/L, while the Cu and Zn values are expressed in mg/L [63].

The Department of Bromatology (MUB) participated in the quality control program for trace element analyses monitored by the National Institute of Public Health, the National Institute of Hygiene, and the Institute of Chemistry and Nuclear Physics (Warsaw, Poland). The concentrations of Cd, Pb, Cu, Zn, and Se in whole blood and serum were calculated in mmol/L to assess the dyshomeostasis of these metals. The concentrations of mineral components were estimated, and the molar ratios between essential trace elements and toxic metals were also calculated and compared between AIS patients and control subjects using Excel software, based on previous studies [59] on the concentration of antioxidant elements (Se, Zn, Cu). Furthermore, selected serum levels of elementary biochemical factors were determined in the accredited Biochemical Clinical Laboratory of the Medical University of Bialystok Clinical Hospital and compared to laboratory reference values. The fasting lipid profile of each patient, including the values for low-density lipoprotein cholesterol LDL-C, total cholesterol TC, triglycerides TG and high-density lipoprotein cholesterol (HDL-C), was evaluated using enzymatic methods and expressed in mg/dL.

### 2.2. Statistical Methods

The frequency of qualitative variables was contrasted between the groups using Pearson’s χ^2^ test. Mann–Whitney tests were used to determine disparities between the groups for continuous variables. The correlations between continuous or ordinal variables were estimated using Spearman’s rank correlation coefficients. Multivariable models for continuous outcomes were identified using generalized linear models. Statistics-related calculations were performed utilizing IBM SPSS Statistics 26.0 software [64].

## 3. Results

We examined 187 sequential AIS patients, including 85 patients who received interventional management and 102 patients who underwent conservative treatment, and compared them to 94 control subjects. There was no statistically significant difference in the distribution of males and females between the AIS patients and the control subjects (*p* = 0.058). Over 90% of AIS patients had arterial hypertension, and a higher mean body mass index (BMI) compared to the control subjects (27 vs. 25.2, *p* < 0.05).

As indicated by the clinical findings, brain lesions were more prevalent in the left hemisphere of the brain, within the anterior cerebral circulation (77%). It was observed that a high proportion (over 79%) of AIS patients exhibited unexpected results on extracranial carotid Doppler ultrasound (characterized by a protrusion of carotid intima–media thickness (CIMT) of more than 1.5 mm in the lumen or intensified thickening of the middle layer of the focal area of more than 50% of the area encompassing the vessel). This was particularly prevalent in the left internal carotid artery. Carotid atherosclerotic plaques with low echogenicity were found in 99 (53%) of AIS patients (including mixed echogenicity in 71 AIS patients). The baseline demographic characteristics, the biochemical values, the levels of Pb and Cd in whole blood, and the levels of Cu, Zn, and Se in serum in AIS patients and control subjects are shown in Table 1 and Table 2.

**Figure 1:**
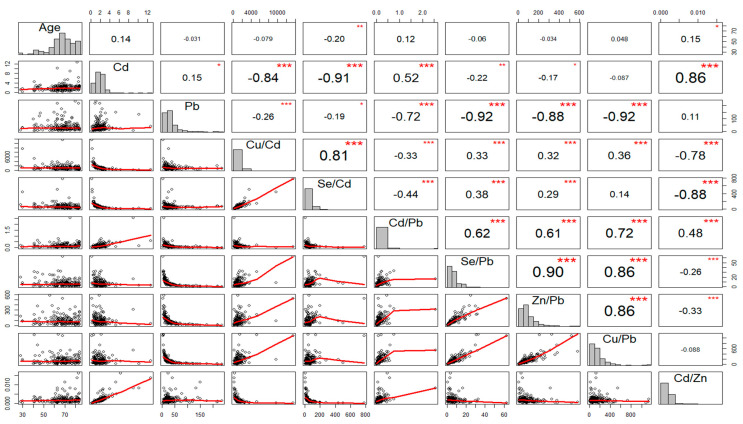
Scatter-matrix, histogram, and Spearman rank correlation matrix of essential trace elements, toxic elements and molar-ratio levels of the aforementioned components in patients with acute ischemic stroke. *p*-values < 0.05 showed statistical validity; * *p* < 0.05, ** *p* < 0.01, *** *p* < 0.001.

The concentration of Cd in the blood was considerably higher (*p* < 0.0001) in AIS patients as compared to those of control subjects. We found that the molar ratios of Cd/Zn and Cd/Pb were significantly higher (*p* < 0.001; *p* < 0.001, respectively), when the molar ratios of Se/Pb, Se/Cd, Cu/Cd were significantly lower (*p* = 0.01; *p* < 0.001; *p* < 0.001, respectively), in AIS patients, as compared to control subjects. However, there were no significant fluctuations in relation to the blood Pb concentration (*p* = 0.223) or molar ratios of Zn/Pb and Cu/Pb (*p* = 0.059; *p* = 0.898, respectively), between AIS patients and control subjects (Table 2). Interestingly, most of the toxic and essential trace elements studied were correlated with each other (Figure 1).

In this dissertation, we explored the potential relationships between the concentration of trace elements in plasma and whole blood and the traditional risk factors for stroke. Our investigation revealed that statistically prominent correlations were found between fibrinogen levels and the Cd and Cd/Zn molar ratios. At the same time, an inverse relationship was observed between fibrinogen levels and the Se/Cd molar ratio. Furthermore, we were able to observe positive correlations between D-dimer levels and Cd/Pb and Cu/Pb molar ratios. Additionally, the Cu/Cd molar ratio had a significant association with the NIHSS score on admission (r = 0.18, *p* = 0.016) and C-reactive-protein (CRP) levels (r = 0.15, *p* = 0.045). In AIS patients, we found positive correlations between uric-acid levels and the Cd/Pb molar ratio, as well as between the levels of the N-terminal prohormone of the brain natriuretic peptide (Nt-proBNP) and the Se/Cd and Cd/Pb molar ratios. These findings suggest the existence of potential associations between concentrations of trace elements and stroke risk factors, but comprehensive research seems to be essential to fully understand the underlying mechanisms.

The study revealed statistically significant differences in the Cu/Pb molar ratios among AIS patients according to their etiology classified by the TOAST classification. The group with LAA etiology had lower Cu/Pb molar ratios as compared to the CE- and SVO-etiology groups (*p* = 0.029). Furthermore, the type of treatment received (interventional therapy versus conservative treatment) influenced the parameters studied in AIS patients. Patients who received conservative treatment had lower Cu/Cd and Se/Cd molar ratios as compared to those who received interventional therapy (*p* = 0.007 and *p* = 0.028, respectively). Furthermore, AIS patients with a more advanced stage of ICA atherosclerosis had higher NIHSS scores on admission and discharge and higher mRS scores on discharge (*p* = 0.003, *p* = 0.005, and *p* = 0.012, respectively). No significant correlations were found between BMI and the toxic elements studied in AIS patients. Multiple associations between clinical variables examined in AIS patients were identified, indicating that ischemic stroke has a complex etiology.

**Figure 2:**
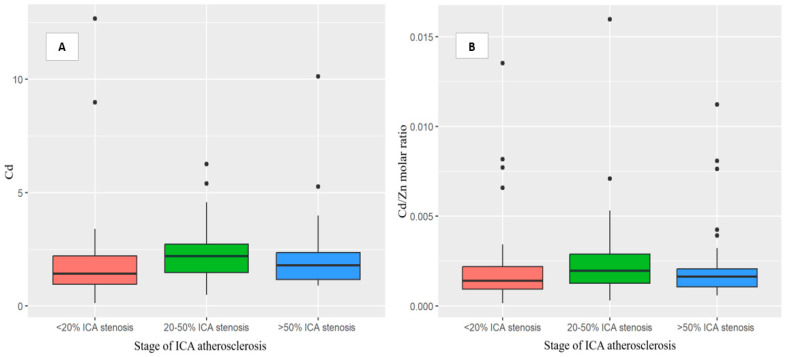
Box plot showing the findings regarding the Cd (**A**) and Cd/Zn (**B**) molar-ratio levels in acute-ischemic-stroke patients in relation to the stage of ICA atherosclerosis—B-mode ultrasound imaging using a standardized protocol or neck CT angiography. The patients with more advanced stages of ICA atherosclerosis had higher concentrations of the Cd and Cd/Zn molar-ratio levels, particularly those with 20–50% ICA stenosis. Abbreviations: Lead, Pb. Cadmium, Cd. Copper, Cu. Selenium, Se. Zinc, Zn. Internal carotid artery, ICA. Computed tomography, CT. Acute ischemic stroke, AIS. *p*-values < 0.05 showed statistical validity.

Furthermore, a positive correlation was found between the stage of ICA atherosclerosis assessed by Doppler ultrasound and Cd levels (r = 0.24, *p* = 0.001), and the Cd/Zn molar ratio (r = 0.15, *p* = 0.037). On the other hand, the stage of ICA atherosclerosis had negative correlations with the Cu/Cd and Se/Cd molar ratios (r = −0.18, *p* = 0.014; r = −0.23, *p* = 0.002, respectively). However, after adjusting for smoking status, these correlations were no longer statistically significant (*p* > 0.05) (Table 3). No correlation was observed between the Zn and Cd concentrations (r = −0.04; *p* = 0.59). We also found that patients with ICA atherosclerosis, particularly those with 20–50% ICA stenosis, had higher concentrations of Cd and Cd/Zn (Figure 2) but lower Cu/Cd and Se/Cd molar ratios. Furthermore, our results demonstrated an association between serum lipid profile and trace elements. Specifically, only the molar ratios of Zn/Pb and Cd/Zn were significantly correlated with HDL values (r = −0.16, *p* = 0.031, r = 0.16, *p* = 0.027, respectively). At the same time, no significant associations were observed between LDL, TG, TC, non-HDL, and toxic elements.

No statistically significant correlations were found between the toxic elements studied and the size of brain lesions, the NIHSS and mRS scores on discharge, ejection fraction (EF), creatinine levels, homocysteine levels, hemoglobin levels or hemoglobin A1C levels (*p* > 0.05). Additionally, there were no statistically significant differences in the levels of toxic metals based on the location of the brain lesion, the presence of type 2 diabetes mellitus, atrial fibrillation, hypertension, hyperlipidemia, or the type and location of atherosclerotic plaques in AIS patients (*p* > 0.05).

In the course of our analysis, we observed that current smokers among AIS patients had significantly higher blood Cd concentrations, Cd/Zn and Cd/Pb molar ratios, and hemoglobin levels, but significantly lower HDL-C concentrations, Se/Cd, and Cu/Cd molar ratios. In contrast, no significant variations were found regarding the concentration of Pb (*p* = 0.702). We also found that current smokers were more likely to have LAA etiology, while never-smokers were more likely to have a CE etiology (*p* = 0.014). In terms of the stage of ICA atherosclerosis, current smokers had a higher prevalence of advanced stages evaluated by Doppler ultrasound examination (*p* < 0.0001). Higher Cd concentrations divided into quartiles were also observed in current smokers (*p* < 0.001) and patients with higher stages of ICA atherosclerosis assessed by Doppler ultrasound (*p* = 0.018). In fact, more than 80% of AIS patients in quartiles 3 and 4 of blood-Cd concentrations (>1.568 µg/L) were current smokers, while only 40.4% of never-smokers were in these quartiles. There were no statistically relevant links between blood-Cd quartile levels and variables such as sex, age, type 2 diabetes mellitus, fasting lipid profile, CRP and homocysteine values.

A generalized linear regression model showed that factors such as current smoking status, atrial fibrillation, advanced age, and lower NIHSS scores on admission significantly increase Cd concentrations in AIS patients. Furthermore, a lower BMI index, advanced age, and current smoking status were significant predictors of elevated Cd/Zn molar-ratio concentrations. Conservative treatment applied in AIS patients, a more severe stage of ICA atherosclerosis, and higher hemoglobin values were related to higher Pb concentrations in these patients (Table 4A–C).

We observed a prominent correlation between age and Cd/Zn molar levels, and a negative correlation between age and Se/Cd molar ratios in AIS patients (r = 0.15; *p* = 0.042, r = −0.20; *p* = 0.005, respectively). In control subjects, lower Se/Pb and Cu/Pb molar ratios were observed in older patients (r = −0.21, *p* = 0.0047; r = −0.25, *p* = 0.016, respectively). Furthermore, significant differences in Cu/Cd molar levels were found between male and female AIS patients (*p* = 0.0033). Lastly, female control subjects had significantly higher levels of the Cu/Pb molar ratio as compared to males (*p* = 0.001) (Table 2).

## 4. Discussion

At present, few, if any, detailed investigations have been conducted to analyze the status of toxic metals in AIS patients in Podlaskie Voivodeship, Poland. The present study’s results have crucial public health implications, highlighting the presence of an imbalance of trace elements in patients with acute ischemic stroke.

The most significant observation in our study was that higher concentrations of Cd in the blood had a crucial impact on the development of ischemic stroke, even at exposure levels that are relatively low. We found that the molar ratios of Cd/Zn and Cd/Pb were significantly higher (*p* < 0.001; *p* < 0.001, respectively), when the molar ratios of Se/Pb, Se/Cd, Cu/Cd were significantly lower (*p* = 0.01; *p* < 0.001; *p* < 0.001, respectively), in AIS patients as compared to control subjects. We also found that patients with higher stages of ICA atherosclerosis, particularly those with 20–50% ICA stenosis, had higher Cd and Cd/Zn concentrations but lower Cu/Cd and Se/Cd molar ratios. In this research, current smokers had a higher likelihood of LAA etiology, whereas CE etiology was more likely to be found among never-smokers. In contrast, we did not find substantial differences in the concentration of Pb in the blood between AIS patients and control subjects. There is a significant interest in the detection of heavy metals within medical science, given the mounting evidence supporting the connection between environmental pollutants and stroke. Ongoing experimental and epidemiological research is highlighting heavy metal exposure as a possible risk factor for stroke, with some recent studies suggesting a positive association between higher blood-Cd levels and stroke prevalence [5,11,14,32,35,39,40,41,42,65,66]. The results of a systematic review and meta-analysis published recently suggest that chronic exposure to Pb, Cd, and Cu could be linked to an elevated risk of stroke [5]. The systematic review by Dev et al. [66] did not provide sufficient evidence to either support or dismiss the relationship between heavy metal exposure and ischemic stroke. However, in our present study, there were no significant fluctuations in relation to blood-Pb concentration in AIS patients, which was also in line with other studies [11,54,67]. It should be pointed out that previous epidemiological studies have produced contradictory findings regarding the correlation between blood-Pb levels and ischemic stroke, findings that align with those of the present study.

Most epidemiological studies investigating the link between toxic metals and ischemic stroke have been limited to examining one metal and various methods to measure its concentration. Recent investigations [14,34,68] have presented a novel approach for assessing the risks associated with multiple metals, with an emphasis on the fact that the general public is exposed to numerous metals in daily life [69]. The extensive prevalence of toxic metals in the environment, coupled with the limitations of current analytical techniques and other factors, makes it difficult to set appropriate health-based thresholds for some of these metals. Yet, recent advancements in nanotechnology and sensor technologies have become essential enablers for detecting toxic trace elements [70].

The atherogenic mechanism of Cd may be associated with oxidative stress, inflammation, endothelial dysfunction, and increased lipid synthesis, which may lead to the inhibition of vascular smooth-muscle-cell proliferation [30,50,51,71,72]. Some studies have found that Cd levels within symptomatic carotid atherosclerotic plaques are 50 times higher than within the blood, and concentrations are the highest in the areas of the plaque where ruptures tend to occur [27]. Population studies conducted in Sweden have also revealed a substantial association between Cd levels in the blood and the presence of the soluble urokinase plasminogen activator receptor, a biomarker related to the atherosclerotic process [51,73]. Therefore, the current study’s findings affirm that elevated Cd levels are related to internal carotid atherosclerosis, which has been corroborated by other studies [14,31,35,74]. The Borné et al. study [24] postulates that Cd may be involved in plaque rupture mechanisms. This hypothesis is supported by the fact that exposure to Cd has been linked to prothrombotic and antifibrinolytic effects. In addition, sex (female) and smoking have been identified as risk factors for plaque erosion, and these risk factors are associated with high exposure to Cd in the general population [23]. Although individuals’ blood-Cd concentrations vary according to age, sex, diet, residential area, and smoking status, exposure to Cd may explain disparities in stroke rates due to regional diversity and, thus, differences in levels of exposure among humans [69,71]. The positive alterations observed in the concentrations of the Cd and Cd/Zn molar ratios in our analysis need further medical research to assess if they can be used as standalone biomarkers of atherosclerosis in AIS patients. The presence of plaque and high levels of Cd appears to be linked to both causes of stroke, LAA and SVO [35,75]. Our study revealed statistically significant differences only in the Cu/Pb molar ratios among AIS patients, based on their etiology as classified by the TOAST classification. The group with LAA etiology had lower Cu/Pb molar ratios as compared to the CE- and SVO-etiology groups. Our study revealed statistically significant differences only in Cu/Pb molar ratios among AIS patients, based on their etiology as classified by the TOAST classification. The group with LAA etiology had lower Cu/Pb molar ratios as compared to the CE- and SVO-etiology groups.

The impact of smoking on Cd levels in the urine and blood is well-documented [76,77,78,79]. After factoring in smoking habits, most studies indicated significant associations between Cd with carotid artery plaque or intima-media thickness (cIMT) of the carotid artery [23]. The studies mentioned above [14,26,30,32,80] provide strong evidence for the link between Cd exposure and risk of stroke, even after adjusting for smoking status. Other investigations have tried to solve this dilemma through subanalyses limited to non-smokers. Nonetheless, they have arrived at conflicting conclusions, ranging from no correlation to connections discovered between individuals who smoke and those who do not smoke [14,25,25,27,37,38,41,65]. In our study, current smokers among AIS patients were characterized by higher blood-Cd concentrations, Cd/Zn and Cd/Pb molar ratios, and hemoglobin values, but lower HDL-C concentrations, Se/Cd, and Cu/Cd molar ratios. Furthermore, we observed a positive correlation between the stage of ICA atherosclerosis in Doppler ultrasound examination and the Cd and Cd/Zn molar ratios, but a negative correlation with the Cu/Cd and Se/Cd molar ratios. However, no correlations were observed after adjustment for smoking status. It is worth noting that Farenberg et al. discovered that blood-Cd levels had a positive correlation with smoking status, age, serum TG, HbA1c levels, and high-sensitivity C-reactive-protein (hsCRP) values [31]. Furthermore, the prevalent stroke etiology of current smokers in our analysis was LAA etiology, which was also observed in the Fagerberg et al. study [73]. A generalized linear regression model allowed us to demonstrate that factors such as smoking status (current smokers), the coincidence of atrial fibrillation, advanced age, and lower NIHSS on admission significantly increase Cd concentration in AIS patients. Taking smoking into account does not rule out the possibility of residual confounders, yet a large number of studies carried out on never-smokers have found a connection between Cd and ASCVD (atherosclerotic cardiovascular disease) above B-Cd >0.5 μg/L [23]. In never-smokers, diet is the main source of Cd. In smokers, it is becoming increasingly plausible that Cd may, in part, mediate the risk of smoking on ASCVD [74]. The risk of myocardial infarction and ischemic stroke associated with smoking seems to disappear within 5 years of quitting [81]. Furthermore, a cross-sectional study in China indicated that the risk of carotid plaques decreased after 10–19 years of not smoking [82].

The review of recently published medical publications has shed new light on the possibility of interactions between essential elements and toxic metals, such as Cd-Zn. Both deficiency and excess of essential and toxic metals can impair immune functions and inevitably lead to CVD [83,84,85]. Exposure to Cd has been reported to increase the likelihood of the development of atherosclerosis. In contrast, the administration of Zn has the opposite effect in reliable rabbit and mouse models of atherosclerosis [76]. Zn, considered an essential metal, has been found to reduce the negative impacts of Cd [69,86]. The research carried out by Chen et al. found that never smoking and maintaining high serum-Zn levels could potentially mitigate the adverse effects of Cd [41]. Furthermore, some studies have indicated that lower levels of Zn were present in smokers, while other investigations revealed that Zn levels were not significantly affected by smoking [76]. It should be noted that this pattern was observed in our study. However, we did not observe any correlations between Zn and Cd concentrations in AIS patients. Cd and Pb have characteristics similar to those of Zn, and can compete for protein metal-binding sites. Some essential metals, such as Zn, can reduce the intestinal absorption of Cd and Pb, restore homeostasis in the body, and reduce the oxidative stress caused by Cd and Pb [87]. In our previous study, we detected a significant drop in serum Zn and Se levels with elevated concentrations of Cu/Se and Cu/Zn molar ratios in AIS patients. This can probably be attributed to the intense inflammatory state and oxidative stress caused by ischemic stroke [59].

Our previous investigation revealed that individuals with AIS and dyslipidemia had higher Se levels. The modifications observed in Cu, Se, and Zn concentrations require additional study to determine their value as independent biomarkers of atherosclerosis in AIS patients [59]. Surprisingly, only the Zn/Pb and Cd/Zn molar ratios were significantly correlated with HDL levels in the current study, while no significant associations were observed between LDL, TG, TC, non-HDL and toxic elements in AIS patients. The results of numerous studies conducted among humans and animals, and experimental studies show that high exposure to Cd may promote hyperlipidemia, which is characterized by elevated levels of TG, TC, and LDL-C, and decreased HDL-C [23,30,33,40,88,89,90,91,92,93,94]. Furthermore, exposure to Cd and Pb was closely associated with atherogenic changes in a lipid profile [30,89,90,91,94,95,96,97].

The studies conducted by Messner et al. (2009) and Rogalska et al. (2009) have shown that exposure to Cd can lead to oxidative stress, inflammation, and the impaired functioning of the lining of blood vessels in experimental models [71,98]. Numerous studies have indicated that higher levels of Cd in the blood and urine are associated with a higher risk of inflammatory complications, as evidenced by increased CRP levels in the blood [23,51,99]. This elevation in inflammation is believed to increase the risk of CVD [33,100]. Our analysis revealed that a higher Cu/Cd molar ratio was associated with elevated CRP values. In addition, Cd and Pb have been associated with changes in the coagulation profile, especially fibrinogen levels [85,96,97]. In our study, it was also revealed that there is a significant association between the toxic elements studied and hemostasis parameters.

Moreover, various investigations have indicated that the coexistence of Cd exposure and obesity could have a notable influence on the occurrence of prediabetes [52]. The impact of metal pollutants on obesity has been demonstrated through their modulation of adipogenesis and the functioning of adipose tissue [54]. Furthermore, various studies have linked multiple exposures to metals to coronary heart disease and obesity [101,102]. Furthermore, a compilation of metal exposures (Pd, Cd, Hg, As) has been shown to be related to obesity and its associated conditions, such as type 2 diabetes mellitus and hypertension [103]. However, there were no significant correlations between BMI index, type 2 diabetes mellitus, and the toxic elements studied in our studied group of AIS patients.

The Global Burden of Diseases Study (GBD) reported that exposure to Pb accounted for approximately 5% of stroke-related deaths and DALYs in 2019 [104]. There is increasing evidence that Pb toxicity is associated with increased oxidative stress due to ROS formation (reactive oxygen species), depletion of antioxidant capacity and increased lipid peroxidation [105,106,107]. Clinical research has revealed that high and low blood levels of Pb can adversely affect cardiovascular health, resulting in an increased risk of cardiovascular diseases, such as stroke [32,54,55,58,95,96,97,108,109,110,111,112,113]. In order to establish a link between trace elements and stroke, Medina Estévez et al. [113] evaluated 45 elements and found that Pb was positively associated with ischemic stroke in both univariate and multivariate analyses. However, there were no considerable fluctuations in relation to the blood-Pb concentration in our AIS patients, which was also in line with other similar studies [11,54,67]. Furthermore, long-term exposure to Pb may be correlated with a possible risk of ICA atherosclerosis [97].

The primary purpose of this preliminary evaluation was to measure the concentrations of toxic blood trace elements in AIS patients in Poland, to broaden our understanding of the broad distribution and mortality of ischemic stroke. Our findings go beyond those of previous research, by providing additional evidence of the relationship between toxic trace elements, ischemic stroke, and atherosclerosis, as well as establishing a basis for conventional behavioral risk factors such as smoking and an unhealthy diet. Unlike previous investigations that mostly focused on a single heavy metal level, our research also examined the cumulative effects of multiple essential and toxic trace elements. Evaluating metal mixtures is essential, as these components coexist [114]. Similarly, future research should address the role of common exposures in atherosclerosis. To assess cardiovascular risk through alterations in environmental metals, prospective studies with multiple observations over a period of time are needed. Repeated assessments of multiple metal exposures must be performed to establish critical susceptibility windows. Thus, the discrepancies observed in the presented studies could, to some extent, be attributed to the exposure to metal mixtures that may concurrently contribute to the occurrence of ischemic stroke. It should be noted that this research was limited, since it was carried out in one department, and only one-time data were available from each participant. A single blood/urine sample is insufficient to accurately reflect the total body burden of toxic trace elements in a given region, and thus cannot be taken as an indicator of the epidemiological condition of the population across different geographical regions and varying exposure levels. The study was unable to track toxic element levels before the event of ischemic stroke. Subsequently, it was impossible to demonstrate particular changes in the levels of toxic elements in the blood over a longer time. Smoking status, sex, and age are essential covariates that must be considered when examining the influence of metal mixtures on the chance of developing CVD and stroke [69]. Despite accounting for various confounding factors, it is still conceivable that significant confounding factors, such as exposure to tobacco, were omitted or inadequately taken into consideration. Moreover, due to the limited data, it was not possible to adjust for pack-years of smoking. Furthermore, the self-reported covariates (smoking status) may have induced recall bias. Despite accounting for various confounding factors, it is still possible that significant confounding factors, such as tobacco exposure, were omitted or inadequately considered. Furthermore, due to limited data, it was not possible to adjust for pack-years of smoking. Furthermore, self-reported covariates (smoking status) may have induced recall bias. Despite the elimination of potential confounders in the analysis, the duration of exposure to heavy metals remains uncertain. Consequently, more research needs to be conducted, focusing on never-smokers or collecting more detailed information on tobacco use, including quit-years of former smokers, smoking traces, or biomarkers associated with tobacco users (e.g., nicotine levels). The confounding impact of smoking may not have been properly accounted for, due to our method of measuring exposure, which was accomplished through blood-Pb level, which captures only recent exposure to lead and therefore does not allow cumulative or long-term exposure to be evaluated. Thus, it was uncertain whether the results seen in this study were related to recent or chronic exposure to Pb.

## 5. Conclusions

Our research showed that the disturbance of the metal balance, Cd, in particular, plays a crucial role in the pathogenesis of AIS. Furthermore, our results expand on those of previous studies on exposure to Pb and Cd as risk factors for AIS. The findings of the study reinforce the hypothesis that both smoking and blood-Cd concentrations are connected with the incidence of AIS. It is critical to investigate the potential role of exposure to metal mixtures in AIS. An accurate assessment of changes in the molar ratios of essential and toxic trace elements could serve as a valuable indicator of the nutritional status and levels of oxidative stress in AIS patients. The Cd/Zn molar ratio has the potential to serve as a useful biomarker for atherosclerosis in AIS patients. With heavy metal exposure being a critical public health issue worldwide, identifying heavy metal pollutants can play a pivotal role in predicting stroke and devising appropriate primary and secondary prevention and control strategies. Additional research is required to fully understand the impact of exposure to essential and toxic trace elements on the potential mechanisms underlying the development of ischemic stroke. Future studies should seek to establish the optimum levels of essential trace elements in the body and devise effective dietary strategies that can mitigate the risk of stroke resulting from toxic metals.

## Figures and Tables

**Table 1:** Descriptive statistics for demographic data and biochemical values of study population.

Clinical Parameters	AIS Patients (n = 187)	Controls (n = 94)	*p* *
Gender (M n (%)/F n (%))	96 (51.3%)/91 (48.7%)	37 (39.4%)/57 (60.6%)	0.058
Age (years) median (Q1–Q3)	69 (63;76)	56.5 (40;65)	<0.05
BMI (Kg/M^2^) median (Q1–Q3)	27 (24.6; 30.4)	25.2 (22.9; 27.8)	<0.05
Total cholesterol (TC) (mg/dL) median (Q1–Q3)	185.5 (150;219.3)	**	**
Triglyceride (TG) (mg/dL) median (Q1–Q3)	106 (79;137.8)	**	**
Low-density lipoprotein cholesterol (LDL-C) (mg/dL) median (Q1–Q3)	122 (92,156.3)	**	**
High-density lipoprotein cholesterol (HDL-C) (mg/dL) median (Q1–Q3)	47 (38;56)	**	**
Hypertension n (%)	166 (88.8%)	**	**
Diabetes mellitus n (%)	65 (34.8%)	**	**
Smokers n (%)	83 (44.4%)	**	**
Obese n (%) BMI ≥ 25	133 (71%)	51 (54.3%)	**
C-reactive protein (CRP) (mg/L) median (Q1–Q3)	3.1 (1.5;6.2)	**	**
Brain lesion size (mm^2^) median (Q1–Q3)	440 (169;1225)	**	**
Lesion location (R n(%)/L n(%) hemisphere)	80 (42.8%)/93 (49.7%), both 14 (7.5%)	**	**
NIHSS on admission median (Q1–Q3)	8 (6;12)	**	**
NIHSS at discharge median (Q1–Q3)	3 (1;5)	**	**
MRS scale median (Q1–Q3)	2 (0;6)	**	**
HbA1c (%) median (Q1–Q3)	5.9 (5.6;6.3)	**	**
Creatinine (mg/dL) median (Q1–Q3)	0.85 (0.73;1)	**	**
Highly sensitive troponin (ng/L) median (Q1–Q3)	10 (10;12.2)	**	**
Fibrinogen (mg/dL) median (Q1–Q3)	370 (320;437)	**	**
D-dimer (µg/mL) median (Q1–Q3)	0.8 (0.4;1.5)	**	**
Uric acid (mg/dL) median (Q1–Q3)	5.3 (4.2;6.9)	**	**
Hemoglobin (g/dL) median (Q1–Q3)	13.7 (12.8;14.8)	**	**
Homocysteine (µmol/L) median (Q1–Q3)	11 (8.9;14.7)	**	**
Intervention treatment (T ± MT) n (%)	85 (45.5%)	**	**
Trombolysis (T) n (%)	73 (39%)	**	**
Mechanical thrombectomy (MT) n (%)	32 (17.1%)	**	**
Hyperlipidemia n (%)	146 (78.5%)	**	**
Atrial fibrillation n (%)	62 (33.2%)	**	**
Carotid atherosclerosis	148 (79.1%)	**	**
>30% stenosis n (%)	47 (25.2%)	**	**
TOAST classification	187 (100%)	**	**
LAA n (%)	69 (36.9%)	**	**
SVO n (%)	42 (22.5%)	**	**
CE n (%)	76 (40.6%)	**	**

Abbreviations: Acute ischemic stroke, AIS. Number, n. Body Mass Index, BMI. Female, F. Male, M. Left, L. Right, R. Large artery atherosclerosis, LAA. Small vessel occlusion, SVO. Cardioembolism, CE. Descriptive data are shown as numbers (percentage) for nominal variables and median (1st–3rd quartile) for quantitative variables. * The Mann–Whitney Test *p*-value. ** Descriptive analysis (control group) could not be performed, due to the unavailability of data.

**Table 2:** The comparison of trace elements status between AIS and controls.

		AIS Patients	Controls	*p* *
Cd [μg/L] [63]	Total	1.8 (1.1–2.5)	1.2 (0.8–1.9)	<0.001
	Males	1.9 (1.2–2.5)	1.2 (0.7–2)	
	Females	1.7 (1–2.4)	1.2 (0.9–1.6)	
	*p* **	0.128	0.786	
Pb [μg/L] [63]	Total	26.2 (16.8–42.3)	31.3 (19.6–46.3)	0.223
	Males	27.4 (18.2–41.1)	36.8 (23.7–48.1)	
	Females	25.1 (14.4–43)	27.2 (17.7–43.2)	
	*p* **	0.274	0.064	
Cd/Zn molar ratio ***	Total	0.0016 (0.0010–0.0024)	0.0009 (0.0005–0.0014)	<0.001
	Males	0.0016 (0.0011–0.0027)	0.0010 (0.0005–0.0015)	
	Females	0.0014 (0.0010–0.0023)	0.0008 (0.0006–0.0013)	
	*p* **	0.329	0.911	
Cu/Cd molar ratio ***	Total	968.6 (621–1447.2)	1680 (829.7–2376.4)	<0.001
	Males	909.3 (553–1396)	1247.1 (829.7–2376.4)	
	Females	1073.1 (704.5–1660.2)	1836.3 (1050–2413.4)	
	*p* **	0.033	0.217	
Se/Cd molar ratio ***	Total	44.6 (30–75.3)	88.1 (55.1–141.1)	<0.001
	Males	42.7 (26.5–70.1)	78.9 (54.8–182.3)	
	Females	49.1 (30.8–85.8)	92.6 (57.1–132.8)	
	*p* **	0.189	0.911	
Cd/Pb molar ratio ***	Total	0.12 (0.07–0.19)	0.08 (0.05–0.13)	0.001
	Males	0.12 (0.07–0.18)	0.07 (0.04–0.12)	
	Females	0.11 (0.07–0.20)	0.08 (0.05–0.14)	
	*p* **	0.802	0.056	
Se/Pb molar ratio ***	Total	5.7 (3.2–9)	6.5 (4.6–10.4)	<0.001
	Males	5.2 (3–8.6)	6.2 (4.2–8.4)	
	Females	6.2 (3.4–9.2)	6.7 (4.8–12.4)	
	*p* **	0.236	0.108	
Zn/Pb molar ratio ***	Total	71.4 (45.6–126.8)	87 (53.4–146.2)	0.059
	Males	76.9 (39.7–112.9)	73.1 (52.6–99.2)	
	Females	68 (47.6–134.7)	97.1 (54–164.5)	
	*p* **	0.467	0.08	
Cu/Pb molar ratio ***	Total	120.9 (62.3–201)	103.8 (70.9–211.1)	0.898
	Males	105.1 (57.5–176)	85.5 (57.7–130.0)	
	Females	128.2 (69.8–254.7)	134.4 (86.2–241.6)	
	*p* **	0.107	0.001	

Abbreviations: Acute ischemic stroke: AIS. Copper, Cu. Selenium, Se. Zinc, Zn. F, Female. M, Male. Descriptive data are shown as median (1st–3rd quartile). Reference values of Trace Elements [63]: Cd (<1.0 µg/L), Pb (<100 µg/L), Se (66–104 µg/L), Zn (0.7–1.3 mg/L), Cu (0.7–1.6 mg/L). * AIS vs. Controls comparison, Mann–Whitney test. ** Females vs. Males comparison, Mann–Whitney test. *** There are not yet any population-based reference values.

**Table 3:** Correlations between the stage of ICA atherosclerosis and concentration of selected essential TEs, toxic elements and the molar ratios of these trace-element concentrations in AIS patients.

	Stage of ICA Atherosclerosis					
	Total		Smokers		Never-Smokers	
	r	*p*	r	*p*	r	*p*
Cd	0.24	* 0.001	0.08	0.457	0.16	0.095
Pb	0.06	0.423	0.05	0.679	0.05	0.617
Zn	0.11	0.152	0.01	0.920	0.23	* 0.02
Cu	0.02	0.768	0.00	0.999	0.08	0.430
Se	−0.04	0.628	−0.12	0.292	0.06	0.567
Cu/Cd molar ratio	−0.18	* 0.014	−0.10	0.363	−0.06	0.565
Se/Cd molar ratio	−0.23	* 0.002	−0.15	0.168	−0.12	0.218
Cd/Pb molar ratio	0.11	0.150	0.04	0.746	0.03	0.734
Se/Pb molar ratio	−0.08	0.296	−0.09	0.435	−0.04	0.668
Zn/Pb molar ratio	−0.05	0.532	−0.08	0.489	0.02	0.862
Cd/Zn molar ratio	0.15	* 0.037	0.04	0.720	0.02	0.822
Cu/Zn molar ratio	−0.03	0.723	0.00	1.000	−0.11	0.278
Cu/Pb molar ratio	−0.04	0.567	−0.04	0.688	−0.02	0.831

Correlations were found between the internal-carotid-artery atherosclerosis stage and B-mode external-carotid ultrasound imaging using a standardized protocol or neck CT angiography, TEs and molar ratio of these TEs concentrations in acute ischemic stroke patients. Nevertheless, following the adjustment for smoking status, these correlations were exhibited to be not statistically significant (*p* > 0.05). Abbreviations: Internal carotid artery, ICA. Lead, Pb. Cadmium, Cd. Selenium, Se. Copper, Cu. Zinc, Zn. Computed tomography, CT. Trace element, TE. Acute ischemic stroke, AIS. * *p*-values < 0.05 showed statistical validity.

**Table 4:** Generalized linear regression models for Cd (**A**), Pb (**B**), Cd/Zn molar ratio (**C**) in AIS patients.

Cadmium (A)				
Variable	B	95% C.I.		*p*
SEX M (vs. F)	0.037	−0.478	0.552	0.889
Treatment conservative (vs. intervention)	0.060	−0.385	0.505	0.792
Smoking status	1.240	0.784	1.695	* 0.000
Stage of ICA atherosclerosis: >50% ICA atherosclerosis (vs. <20% ICA atherosclerosis)	0.376	−0.212	0.964	0.210
Stage of ICA atherosclerosis: 20–50% ICA atherosclerosis (vs. <20% ICA atherosclerosis)	0.228	−0.254	0.710	0.354
Diabetes mellitus type 2	0.037	−0.438	0.512	0.879
Atrial fibrillation	0.653	0.162	1.144	* 0.009
Age	0.021	0.001	0.041	* 0.038
Brain lesion size (mm^2^)	0.000	0.000	0.000	0.617
NIHSS on admission	−0.067	−0.125	−0.008	* 0.025
BMI index	−0.057	−0.114	0.000	0.050
HDL-C	−0.001	−0.018	0.017	0.931
LDL-C	−0.002	−0.007	0.002	0.276
TG	−0.002	−0.004	0.001	0.142
CRP	−0.045	−0.120	0.031	0.249
Hemoglobin	0.086	−0.073	0.245	0.292
Creatinine	−0.022	−0.811	0.768	0.957
D-dimer	0.016	−0.058	0.091	0.667
Fibrinogen	0.001	−0.002	0.003	0.695
**Lead (B)**				
**Variable**	**B**	**95% C.I.**		***p***
SEX M (vs. F)	−2.760	−18.990	13.470	0.739
Treatment conservative (vs. intervention)	16.920	2.887	30.953	* 0.018
Smoking status	−9.728	−24.092	4.636	0.184
Stage of ICA atherosclerosis: >50% ICA atherosclerosis (vs. <20% ICA atherosclerosis)	25.007	6.480	43.535	* 0.008
Stage of ICA atherosclerosis: 20–50% ICA atherosclerosis (vs. <20% ICA atherosclerosis)	8.593	−6.603	23.788	0.268
Diabetes mellitus type 2	6.990	−7.990	21.969	0.360
Atrial fibrillation	5.503	−9.963	20.969	0.486
Age	−0.257	−0.882	0.368	0.420
Brain lesion size	−0.001	−0.012	0.009	0.791
NIHSS on admission	−0.683	−2.524	1.158	0.467
BMI index	−0.341	−2.137	1.455	0.710
HDL-C	0.325	−0.231	0.882	0.252
LDL-C	0.006	−0.132	0.143	0.937
TG	−0.053	−0.120	0.014	0.121
CRP	−0.107	−2.497	2.284	0.930
Hemoglobin	5.946	0.932	10.961	* 0.020
Creatinine	17.904	−6.976	42.784	0.158
D-dimer	−1.486	−3.847	0.875	0.217
Fibrinogen	0.026	−0.055	0.106	0.533
**Cd/Zn molar ratio (C)**				
**Variable**	**B**	**95% C.I.**		***p***
SEX M (vs. F)	−0.00016	−0.00091	0.00059	0.680
Treatment conservative (vs. intervention)	0.00029	−0.00036	0.00093	0.388
Smoking status	0.00158	0.00092	0.00224	* 0.000
Stage of ICA atherosclerosis: >50% ICA atherosclerosis (vs. <20% ICA atherosclerosis)	0.00010	−0.00076	0.00095	0.823
Stage of ICA atherosclerosis: 20–50% ICA atherosclerosis (vs. <20% ICA atherosclerosis)	0.00033	−0.00037	0.00103	0.354
Diabetes mellitus type 2	0.00019	−0.00050	0.00088	0.595
Atrial fibrillation	0.00055	−0.00016	0.00126	0.131
Age	0.00003	0.00000	0.00006	* 0.032
Brain lesion size (mm^2^)	0.00000	0.00000	0.00000	0.817
NIHSS on admission	−0.00003	−0.00012	0.00005	0.443
BMI index	−0.00011	−0.00019	−0.00003	* 0.010
HDL-C	0.00000	−0.00002	0.00003	0.769
LDL-C	0.00000	−0.00001	0.00000	0.169
TG	0.00000	0.00000	0.00000	0.368
CRP	−0.00008	−0.00019	0.00003	0.172
Hemoglobin	0.00022	−0.00001	0.00045	0.062
Creatinine	0.00036	−0.00078	0.00151	0.535
D-dimer	−0.00001	−0.00012	0.00010	0.854
Fibrinogen	0.00000	0.00000	0.00000	0.814

Abbreviations: Female, F. Male, M. Body Mass Index, BMI. Internal carotid artery, ICA. Acute ischemic stroke, AIS. National Institutes of Health Stroke Scale, NIHSS. Lead, Pb. Cadmium, Cd. Zinc, Zn. High-density lipoprotein cholesterol, HDL-C. Low-density lipoprotein cholesterol, LDL-C. Triglyceride, TG. C-reactive protein, CRP; * *p*-values < 0.05 showed statistical validity.

## Data Availability

All data generated or analyzed during this study are included in this review article.
